# Could breaks reduce general practitioner burnout and improve safety? A daily diary study

**DOI:** 10.1371/journal.pone.0307513

**Published:** 2024-08-27

**Authors:** Louise H. Hall, Judith Johnson, Ian Watt, Daryl B. O’Connor

**Affiliations:** 1 Research Fellow, School of Medicine, University of Leeds, Leeds, United Kingdom; 2 School of Psychology, University of Leeds, Leeds, United Kingdom; 3 Yorkshire Quality and Safety Research Group, Bradford Institute for Health Research, Bradford Royal Infirmary, Bradford, United Kingdom; 4 School of Public Health and Community Medicine, University of New South Wales, Sydney, NSW, Australia; 5 Hull York Medical School, University of York, York, United Kingdom; Kwame Nkrumah University of Science and Technology, GHANA

## Abstract

**Background:**

Rates of burnout are currently at record high levels, and GPs experience higher burnout than many other specialties. Organisational interventions may reduce burnout, but few studies have investigated these in primary care.

**Aim:**

The current study investigated whether breaks, both with and without social interactions, were associated with burnout and patient safety perceptions in GPs.

**Design:**

A within-subjects, interval contingent, quantitative daily diary design.

**Setting:**

UK GP practices.

**Method:**

Participants completed questionnaires at baseline measuring demographic variables, burnout and patient safety perceptions. They then completed a questionnaire in the evening each day for a week which captured whether they had taken a break that day, whether it involved a positive social interaction, burnout (comprising subscales of disengagement and exhaustion), positive and negative affect and patient safety perceptions. The data were analysed using hierarchical linear modelling to assess same-day and next-day associations.

**Results:**

We included 241 responses from 58 GPs for analysis. Taking at least one break (involving any or no social interactions) was associated with lower disengagement that day and lower exhaustion the next day. Taking at least one break involving a positive interaction was associated with 1) lower disengagement, exhaustion, overall burnout and negative affect on the same day, as well as higher positive affect and improved perceptions of patient safety, and 2) lower exhaustion and improved patient safety perceptions on the next day.

**Conclusion:**

Organizing daily team or practice breaks where staff can socialise may help to reduce burnout and improve perceptions of patient safety.

## Introduction

Rates of physician burnout have increased internationally since the onset of the Covid-19 pandemic and are now at their highest on record [1, 2]. Burnout is a syndrome characterised by disengagement and emotional exhaustion, and levels are consistently higher in general practitioners (GPs) than the mean across physicians specialties [3]. A recent UK survey found that 68% of GPs reported high exhaustion and 48% reported high disengagement [4].

Elevated burnout is linked with organizational outcomes including absenteeism, intentions to leave, poorer patient satisfaction and poorer patient safety [5–7]. Interventions for burnout can be categorized as those which are person-directed, aiming to treat an individual without reference to their context, and those which are organization-directed, aiming to improve an individual’s wellbeing by making organizational changes [8]. Person-directed interventions include, for example, mindfulness, stress-management workshops and exercise interventions. Organization-directed interventions include, for example, work-scheduling changes, improved cafeteria facilities and the delivery of professional training. Studies suggest both types of interventions can be effective, but more research has focused upon person-directed interventions, and effect sizes are generally small [8–10].

While organizational interventions may be effective for reducing burnout, few studies have investigated these in primary care settings. One possible intervention is the use of shared breaks. A focus group in GPs indicated this is a feasible intervention which reduces isolation and creates opportunity for positive interactions [11]. However, no quantitative evaluations have been conducted, and in general, there have been a lack of studies in this area. A review of 32 studies into breaks in doctors found only two investigated standard 30-minute breaks [12]. A further two investigated sleep interventions, four investigated yoga and mindfulness, six investigated ‘microbreaks’, seven were cross-sectional survey studies, four were prospective or retrospective cohort studies, four were qualitative studies and one was a mixed-methods study [12]. Only two studies included burnout as an outcome measure, and both investigated yoga or mindfulness rather than general social breaks. Only three of the included studies included primary care physicians, and these did not measure burnout as an outcome measure. Furthermore, despite consistent evidence of a link between burnout and patient safety [13], none of the included studies measured any patient safety outcome indicators [12]. It is also unclear whether it is breaks of any sort that are associated with physician wellness or the opportunity for positive social interactions these can provide.

The present study addressed these issues using a daily diary method, which allows comparisons within individuals across multiple days. We had four key research questions:

1. Is taking a break associated with burnout and wellbeing that day?
2. Is taking a break associated with stress, burnout, and wellbeing the next day?
3. Is taking a break associated with patient safety that day?
4. Is taking a break associated with patient safety the following day?

Within each of these questions, we investigated whether there were associations between breaks of any sort and the outcome variables, and breaks including a positive social interaction.

## Methods

### Design and materials

A within-subjects, interval contingent, daily diary design. Participants completed a questionnaire battery at baseline, followed by a stress questionnaire before work in the morning and a longer questionnaire battery in the evening before bed, for seven consecutive days. Questionnaires were online and participants received reminders via text and email.

### Participants

Practicing UK GPs who worked six sessions a week or more, were eligible.

### Recruitment

Participants were recruited from 1) GP organisations and media outlets, 2)

social media, 3) local practices, 4) local GP continuing professional development events and 5) pre-existing contact lists from prior research and personal network between 1 August 2017 and 1 January 2018.

### Ethical considerations

This study received ethical approval from the University of Leeds, School of Psychology Ethics Committee (ref #17–0185, date: 27/06/17) and Health Research Authority approval (IRAS # 216260). Participants were fully informed of the types of questions that would be involved in the study prior to providing their written informed consent to take part. They were also informed that they may skip any questions they wished to, and that they may withdraw from the study at any point before, during, and after taking part, up until the point of analysis (one month post study completion). They were also informed that their responses would be kept completely anonymous through the use of a unique participant code, with any contact information stored securely and separately to their participant code and survey responses. Finally, contact information for relevant support/helplines were provided at the end of the study in the event that participants became distressed as a result of the potentially sensitive nature of the study.

### Baseline questionnaire

Demographic variables (age, gender, ethnicity, location) and occupational variables (workload, practice list size, number of hours spent on various work tasks etc.) were measured. The GHQ-12 [14] was used to measure mental distress, with scores above 3 indicating a possible psychiatric disorder [15]. It showed good internal consistency in our sample (α = .820). The Quality of Life (QoL) was used to measure a broader sense of participants’ overall wellbeing [16–18]. The four-item shortened version of the PSS (the PSS-4) captured participants’ stress over the last month [19]. Cronbach’s alpha in our sample demonstrated good internal consistency (α = .809). Burnout was measured using the 16-item Oldenburg Burnout Inventory (OLBI) [20]. This measures burnout on two subscales; Exhaustion, Disengagement. Cronbach’s alpha in our sample demonstrated good internal consistency (α = .878). The Safe Practitioner Measure [21] captured perceptions about safety of personal practice using a single item. This measure has been validated in survey and daily diary study research [22–24]. Practice level support was measured with a visual analogue scale (VAS). This question has been previously used within a GP sample and found to be an important contributor to wellbeing, burnout, and patient safety [23].

### Morning questionnaire

Prior to work, participants were asked to complete one question on stress; “How stressed do you feel this morning?” on a 10-point scale.

### Evening questionnaire

Only data from work days was included; non-work days were excluded from the study.

#### Positive and negative affect

Daily levels of wellbeing were measured using positive and negative affect adjectives (details in S1 Methods in the S1 Data).

#### Burnout

Three items from the OLBI with adapted wording for use at the daily level. An example item is, ‘Today I found new and interesting aspects in my work’. Cronbach’s α = .818 demonstrated good internal consistency.

#### Safety perceptions

The daily version of the safe practitioner measure was used [21]. A previous study which used this found good variability across days [22].

#### Adverse events & near misses

Participants were asked whether they had been responsible for any 1) adverse events (AEs) and 2) near misses (NM) that day. Further questions were asked in order to classify the (potential) outcome of the AE/NM; “Minor reversible patient harm, Minor irreversible patient harm, Major reversible patient harm, Major irreversible patient harm”, the type of AE/NM; “Diagnostic error/near miss, Medication or prescription error, Equipment error, Communication, Monitoring error, Other” and the contributors to them; “System issue, Degree of fatigue, Lapse in concentration, Lapse in judgment, Lack of knowledge, Degree of stress or burnout, Other”. For inferential analyses, AEs and NMs were collapsed and coded as 1 (for either present), and 0 (for neither present).

#### Breaks

Participants were asked how many breaks they had each day, how long each break was, and in how many of the breaks they had positive and negative interactions with colleagues.

### Data preparation and analysis

The data set was created by matching participants’ morning responses to their evening responses. Responses from days off work were excluded for the purposes of this study. Survey time points that were completely missing were deleted, along with their corresponding morning responses (*n* = 8). Survey time points that were completed on the wrong day (i.e. backfilled) were excluded from the analysis (*n* = 16) along with one participant who did not complete any evening surveys. The final number of participants was 58, with 241 daily entries.

For the lagged analyses (to determine next day effects), participants were included if they had completed three or more consecutive days at work. To create the data file, participants’ burnout, wellbeing, stress rating, and safety scores from the subsequent day were mapped onto the previous day’s row, and the final day’s scores from that participant deleted. This resulted in 41 participants with a total of 128 days, giving an average of 3 days per participant.

Hierarchical linear modelling in HLM7 was conducted [25]. This allows the data to be assessed both within and between levels. Relationships can be established at a daily level and more accurate conclusions can be drawn through this type of analysis compared to only between-participant analyses, as each participant essentially acts as their own control. The data from this study was across two-levels. Level 1 contained within-participant variation (e.g. daily burnout) with predictor variables centred around the group mean. Level 2 contained between-participant variability (e.g. baseline levels of burnout) with variables centred around the grand mean [26, 27]. Each model controlled for participants’ age, gender, and years in practice. For further data preparation and analysis information, including the equations used for calculating the Pseudo R^2^ to provide an estimate of effect sizes, see the Supplement (S2 Methods in the S1 Data) [28].

## Results

### Descriptive statistics

Fifty-nine GPs took part. Descriptive data (Table 1 and S1 Table in the S1 Data) was based on the dataset used for same-day analyses (*n* = 58 with missing data imputed). Fifty-one (87.9%) participants scored as having a possible minor psychiatric disorder on the GHQ-12, 50 (86%) were classed as having mild (29%) or severe (57%) exhaustion and 44 (76%) were classed as having mild (40%) or severe (36%) disengagement. The average length of breaks was 13 minutes (median = 20, range = 5–120), and there were more days when GPs took breaks than did not take a break (165 vs. 76, respectively; S2 Table in the S1 Data). We measured the number of breaks with a negative interaction with a colleague, but did not include this in any models as there were not enough for statistical power (only 5% of cases in the same day analyses, and 3.9% in the lagged analyses).

**Table 1:** Descriptive statistics for background and daily variables.

Variable	Mean (s.d.) / %	Range
Age: *Same day analyses*^*a*^	44.40 (9.37)	28–66
*Lagged analyses*^*c*^	44.98 (8.67)	28–61
Gender^*a*^	62.1% Female; 37.9% Male	
Ethnicity^*a*^	82.8% White British; 5.2% Asian British; 3.4% White European; 1.7% White Irish; 1.7% Middle Eastern; 5.2% Other Ethnicity	
Years in practice: *Same day*^*a*^	14.09 (9.40)	0.25–38
*Lagged analyses*^*c*^	14.80 (9.05)	1–38
Job role^*a*^	77.6% Partner; 1.7% Locum; 19% Salaried; 1.7% ‘Other’ (e.g. Trainee)	
Practice list size^*a*^	11793.97 (7759.01)	3000–50000
Partners^b^	5.23 (3.60)	0 (n/a) -18
Average hours p/w^*a*^	45.26 (9.86)	26–70
Patient contact hours p/w^*a*^	27.80 (7.36)	16–56
Patients seen p/d^*a*^	33.59 (9.11)	12–70
Extra roles p/w^*a*^	3.43 (4.86)	0–20
Admin hours p/w^*a*^	15.20 (8.32)	3–45
Antisocial hours p/w^*a*^	11.95 (5.95)	2–30
On call p/m^b^	25.02 (23.85)	0–106.5
Location^*a*^	37.9% Urban; 36.2% Suburban; 22.4% Rural; 3.4% ‘Other’	
Supportive practice: *Same day*^*a*^	6.97 (2.12)	2–10
*Lagged analyses*^*c*^	7.12 (2.22)	2–10
Safe practice: *Same day*^*a*^	2.84 (1.07)	1–5
*Lagged analyses*^*c*^	2.85 (1.06)	1–5
QoL: *Same day*^*a*^	6.33 (1.77)	1–9
*Lagged analyses*^*c*^	6.51 (1.52)	3–9
GHQ-12: *Same day*^*a*^	6.48 (2.86)	0–11
*Lagged analyses*^*c*^	6.12 (2.96)	0–11
GHQ ‘caseness’ ^*a*^	87.9% Probable case; 12.1% No case	
PSS4: *Same day*^*a*^	7.03 (2.61)	0–12
*Lagged analyses*^*c*^	6.56 (2.75)	0–11
OLBI Exhaustion: *Same day* ^*a*^	22.5 (4.13)	13–32
*Lagged analyses*^*c*^	22.02 (4.18)	13–32
OLBI Exhaustion level ^*a*^	13.79% None; 29.31% Mild; 56.9% Severe	
OLBI Disengagement: *Same day* ^*a*^	19.86 (3.85)	10–27
*Lagged analyses*^*c*^	19.73 (4.24)	10–27
OLBI Disengagement level ^*a*^	24.14% None; 39.66% Mild; 36.21% Severe	
OLBI total score: *Same day* ^*a*^	42.36 (7.31)	23–59
*Lagged analysis*^*c*^	41.76 (7.80)	23–59

^*a*^*n* = 58, ^b^*n* = 57, ^*c*^*n = 41*

This is prior to missing data being imputed.

*p/w* = per week, *p/d* = per day, *p/m* = per month, *QoL* = Quality of Life, *GHQ-12* = General Health Questionnaire (12-item), *PSS-4* = Perceived Stress Scale (4-item), *OLBI* = Oldenburg Burnout Inventory

The most common outcome for AEs and NMs was (potential for) ‘minor reversible patient harm’ (92% of AEs, 76% of NM). The most common AE and NM types were medication or prescription (42% and 38%, respectively), followed by communication (25% and 35%, respectively). Lapse in concentration was the most cited contributor for the AE or NM (27%), followed by fatigue (24%) and stress or burnout (15%).

### 1. Is taking a break associated with burnout and wellbeing that day?

#### 1a. Taking a break and burnout

Having a break of any sort (including any interaction type) had a significant negative association with disengagement (Tables 2 and 3). This remained significant when controlling for baseline disengagement (β = -.433, p = .040, pseudo R^2^ = .017). There was no significant association between breaks and total burnout or exhaustion.

**Table 2:** Within-person associations between break variables with wellbeing and burnout outcome variables on the same day.

HLM Effect	Symbol	Coeff	SE	*p—*value	Pseudo R^2^
*Intercept*: OLBI *Level 1 slope*: Breaks—OLBI	β_00_ β_10_	15.271 - .795	1.086 .422	< .001 .065	.034
*Intercept*: OLBI *Level 1 slope*: Positive Int.—OLBI	β_00_ β_10_	15.193 -1.490	1.018 .398	< .001 < .001	.050
*Intercept*: OLBI:D *Level 1 slope*: Breaks–OLBI:D	β_00_ β_10_	6.767 - .590	.533 .211	< .001 .007	.025
*Intercept*: OLBI:D *Level 1 slope*: Positive Int.–OLBI:D	β_00_ β_10_	6.735 - .853	.496 .192	< .001 < .001	.054
*Intercept*: OLBI:E *Level 1 slope*: Breaks–OLBI:E	β_00_ β_10_	8.365 - .219	.677 .268	< .001 .418	.014
*Intercept*: OLBI:E *Level 1 slope*: Positive Int.–OLBI:E	β_00_ β_10_	8.404 - .663	.651 .258	< .001 .013	.023
*Intercept*: Positive Affect *Level 1 slope*: Breaks–Positive Affect	β_00_ β_10_	24.867 1.043	3.672 1.245	< .001 .410	.028
*Intercept*: Positive Affect *Level 1 slope*: Positive Int.–Positive Affect	β_00_ β_10_	25.503 3.734	3.5271.333	< .001 .007	.077
*Intercept*: Negative Affect *Level 1 slope*: Breaks–Negative Affect	β_00_ β_10_	28.772 -3.461	5.723 2.088	< .001 .103	.060
*Intercept*: Negative Affect *Level 1 slope*: Positive Int.–Negative Affect	β_00_ β_10_	28.456 -7.124	5.594 2.068	< .001 .001	.116

Note: Level 1 *n* = 241, statistics when controlling for age, gender, and years in practice only, HLM = Hierarchical linear modelling, β = hierarchical multilevel linear modelling symbol, Coeff = unstandardized coefficient, SE = standard error

OLBI = Oldenburg Burnout Inventory (total score), OLBI:D = Disengagement subscale of OLBI, OLBI:E = Exhaustion subscale of OLBI, Int. = Interaction

**Table 3:** Within-person associations between types of breaks with wellbeing, burnout, and safety outcome variables on the same and next day.

	Breaks per se	Breaks with a positive Interaction
OLBI		
*Same Day*	x	**✓**
*Next Day*	x	x
OLBI:D		
*Same Day*	**✓**	**✓**
*Next Day*	x	x
OLBI:E		
*Same Day*	x	**✓**
*Next Day*	**✓** *	**✓** *
Positive Affect		
*Same Day*	x	**✓**
*Next Day*	x	x
Negative Affect		
*Same Day*	x	**✓**
*Next Day*	x	x
Morning Stress		
*Same day*	**n/a**	**n/a**
*Next Day*	x	x
Safe Practitioner		
*Same day*	x	**✓**
*Next Day*	x	**✓**
PSI		
*Same day*	x	x
*Next Day*	x	x

*Same day analyses*: *n* = *58*, *number of days = 241*. *Next day analyses*: *n = 41*, *number of days = 128*. SP = Safe Practitioner, PSI = Patient Safety Incident, OLBI = Oldenburg Burnout Inventory (total score), OLBI:D = Disengagement subscale of OLBI, OLBI:E = Exhaustion subscale of OLBI. **✓** = significant association in expected direction

* = not significant when controlling for baseline exhaustion scores

Breaks with a positive interaction had a significant negative association with total burnout. This held when controlling for baseline total burnout (β = -.927, p = .032, pseudo R^2^ = .038). When using the subscales of burnout independently, breaks with a positive interaction were significantly negatively associated with both disengagement and exhaustion. Disengagement remained significant when controlling for baseline disengagement scores (β = -.639, p = .001, pseudo R^2^ = .040). Exhaustion was no longer significant when controlling for baseline exhaustion scores (β = -.432, p = .111, pseudo R^2^ = .024).

#### 1b. Taking a break and wellbeing

Having a break of any sort during the day was not significantly associated with positive or negative affect on the same day.

Breaks with a positive interaction(s) were significantly positively associated with positive affect and significantly negatively associated with negative affect. These remained significant when controlling for baseline wellbeing scores (GHQ-12: β = 3.258, p = .010 for positive affect, β = -6.621, p = .002 for negative affect, and QoL: β = 3.45, p = .011 for positive affect, β = -6.603, p = .002 for negative affect).

### 2. Is taking a break associated with stress, burnout, and wellbeing the next day?

#### 2a. Taking a break and next day morning stress

Having a break of any sort, and having a break with a positive interaction both had no association with morning stress the following day (Tables 3 and 4).

**Table 4:** Within-person associations between break variables with stress, wellbeing and burnout outcome variables on the next day.

HLM Effect	Symbol	Coeff	SE	*p*—value	Pseudo R^2^
*Intercept*: OLBI *Level 1 slope*: Breaks—OLBI	β_00_ β_10_	*13*.*232* *-0*.*699*	*1*.*322* *0*.*427*	*<* .*001* *0*.*110*	.004
*Intercept*: OLBI *Level 1 slope*: Positive Int.—OLBI	β_00_ β_10_	*13*.*134* *-0*.*793*	*1*.*299* *0*.*437*	*<* .*001* *0*.*077*	-.002
*Intercept*: OLBI:D *Level 1 slope*: Breaks–OLBI:D	β_00_ β_10_	*6*.*082* *-0*.*124*	*0*.*641* *0*.*314*	*<* .*001* *0*.*694*	.015
*Intercept*: OLBI:D *Level 1 slope*: Positive Int.–OLBI:D	β_00_ β_10_	*6*.*089* *-0*.*248*	*0*.*623* *0*.*299*	*<* .*001* *0*.*412*	-.003
*Intercept*: OLBI:E *Level 1 slope*: Breaks–OLBI:E	β_00_ β_10_	*7*.*158* *-0*.*618*	*0*.*826* *0*.*270*	*<* .*001* *0*.028*	.030
*Intercept*: OLBI:E *Level 1 slope*: Positive Int.–OLBI:E	β_00_ β_10_	*7*.*035* *-0*.*661*	*0*.*839* *0*.*301*	*<* .*001* *0*.034*	.026
*Intercept*: Positive Affect *Level 1 slope*: Breaks–Positive Affect	β_00_ β_10_	*32*.*963* *0*.*254*	*4*.*536* *1*.*692*	*<* .*001* *0*.*881*	.021
*Intercept*: Positive Affect *Level 1 slope*: Positive Int.–Positive Affect	β_00_ β_10_	*32*.*970* *-0*.*532*	*4*.*526* *1*.*509*	*<* .*001* *0*.*726*	.004
*Intercept*: Negative Affect *Level 1 slope*: Breaks–Negative Affect	β_00_ β_10_	*12*.*725* *-2*.*665*	*6*.*475* *1*.*722*	*0*.*057* *0*.*130*	.012
*Intercept*: Negative Affect *Level 1 slope*: Positive Int.–Negative Affect	β_00_ β_10_	*12*.*386* *-2*.*219*	*6*.*402* *1*.*973*	*0*.*061* *0*.*267*	-.005
*Intercept*: Morning Stress *Level 1 slope*: Breaks–Morning Stress	β_00_ β_10_	4.360 -0.297	*0*.*929* *0*.*398*	*<* .*001* *0*.*459*	-.003
*Intercept*: Morning Stress *Level 1 slope*: Positive Int.–Morning Stress	β_00_ β_10_	*4*.*507* *-0*.*483*	*0*.*935* *0*.*497*	*<* .*001* *0*.*337*	.060

Note: Level 1 *n = 41*, *number of days = 128*, statistics when controlling for age, gender, and years in practice only, HLM = Hierarchical linear modelling, β = hierarchical multilevel linear modelling symbol, Coeff = unstandardized coefficient, SE = standard error

OLBI = Oldenburg Burnout Inventory (total score), OLBI:D = Disengagement subscale of OLBI, OLBI:E = Exhaustion subscale of OLBI, Int. = Interaction

* = not significant when controlling for baseline exhaustion scores

#### 2b. Taking a break and next day burnout

Having a break of any sort, and specifically having a break with a positive interaction were both significantly associated with lower exhaustion the following day. However, when controlling for baseline exhaustion, neither remained significant (β = -0.443, *p* = 0.106, pseudo R^2^ = .019; β = -0.474, *p* = .122, pseudo R^2^ = .016, respectively). No significant associations were found for disengagement and overall burnout the following day.

#### 2c. Taking a break and next day wellbeing

Taking a break of any sort, and taking a break with a positive interaction were not associated with positive or negative affect on the following day.

### 3. Is taking a break associated with patient safety that day?

#### 3a. Taking a break and safety perceptions

Breaks with a positive interaction had a significant negative association with safe practitioner scores that day (Tables 3 and 5), meaning they were more able to act as a safe practitioner that day. This association remained significant when controlling for baseline safety perceptions (β = -0.343, *p* = .008, pseudo R^2^ = -.003). Breaks of any sort were not significantly associated with safe practitioner scores.

**Table 5:** Within-person associations between break variables with patient safety outcome variables on the same day.

HLM Effect	Symbol	Coeff	SE	*p—*value	pseudo R^2^
*Intercept*: Safe Practitioner *Level 1 slope*: Breaks—SP	β_00_ β_10_	2.528 - .224	.225 .127	< .001 .083	.001
*Intercept*: Safe Practitioner *Level 1 slope*: Positive Int.—SP	β_00_ β_10_	2.532 - .343	.218 .126	< .001 .008	.001
*Intercept*: PSI *Level 1 slope*: Breaks–PSI	β_00_ β_10_	-1.977 - .339	.749 .379	.011 .375	.205
*Intercept*: PSI *Level 1 slope*: Positive Int.–PSI	β_00_ β_10_	-2.064 - .386	.749 .359	.008 .287	.222

Note: Level 1 *n* = 241, statistics when controlling for age, gender, and years in practice only, HLM = Hierarchical linear modelling, β = hierarchical multilevel linear modelling symbol, Coeff = unstandardized coefficient, SE = standard error, OLBI = Oldenburg Burnout Inventory (total score), Int. = Interaction, SP = Safe Practitioner, PSI = Patient Safety Incident.

#### 3b. Taking a break and safety incidents

Breaks of any sort, and breaks with a positive interaction were not associated with reporting involvement in a patient safety incident that day.

### 4. Is taking a break associated with patient safety on the following day?

#### 4a. Taking a break and next day safety perceptions

Taking a break with a positive interaction was associated with perceptions of safer practice on the following day (Tables 3 and 6). This association remained significant when controlling for baseline safety perceptions (β = -0.697, *p* = .001, pseudo R^2^ = .162). No significant association was found for breaks of any sort.

**Table 6:** Within-person associations between break variables with patient safety outcome variables on the next day.

HLM Effect	Symbol	Coeff	SE	*p—*value	pseudo R^2^
*Intercept*: Safe Practitioner *Level 1 slope*: Breaks—SP	β_00_ β_10_	2.271 -0.305	0.286 0.167	< .001 0.076	.006
*Intercept*: Safe Practitioner *Level 1 slope*: Positive Int.—SP	β_00_ β_10_	2.255 -0.690	0.259 0.199	< .001 0.001	.145
*Intercept*: PSI *Level 1 slope*: Breaks–PSI	β_00_ β_10_	-3.072 -0.125	1.084 0.759	0.007 0.870	.156
*Intercept*: PSI *Level 1 slope*: Positive Int.–PSI	β_00_ β_10_	-3.285 0.282	1.097 0.691	0.005 0.686	.219

*Note*: *Level 1 n = 128*, statistics when controlling for age, gender, and years in practice only, HLM = Hierarchical linear modelling, β = hierarchical multilevel linear modelling symbol, Coeff = unstandardized coefficient, SE = standard error, OLBI = Oldenburg Burnout Inventory (total score), Int. = Interaction, SP = Safe Practitioner, PSI = Patient Safety Incident.

#### 4b. Taking a break and next day safety incidents

Taking a break of any sort, and those with positive interactions, were not associated with reporting patient safety incidents on the following day.

## Discussion

### Summary

This is the first study to quantitatively investigate the association between taking breaks with burnout and patient safety in GPs. Taking at least one break during the working day, involving any type of social interaction (including no interactions), was associated with lower levels of disengagement that day and lower exhaustion on the following day. Taking at least one break involving a positive interaction was associated with lower disengagement, exhaustion, overall burnout and negative affect on the same day, as well as higher levels of positive affect and improved perceptions of patient safety. Taking at least one break involving a positive interaction was also associated with lower exhaustion and improved patient safety perceptions on the following day. Taking breaks was not associated with reported stress levels or risk of patient safety incidents, and it should be noted that the next-day associations between breaks and exhaustion were not maintained when controlling for baseline emotional exhaustion.

### Strengths and limitations

A strength of the study was its use of a daily diary study approach, which enabled the analysis of associations both within the same day and across days, and enabled participants to act as their own controls. It may have been limited by a possible response bias, which could have caused physicians who were burnt-out to be more likely to want to respond to study advertisements.

### Comparisons with existing literature

The present study addresses concerns about the erosion of breaks in healthcare settings [29] by reporting the first quantitative evidence of an association between taking breaks, burnout and patient safety perceptions in doctors. A previous systematic review of the literature on breaks and wellbeing in doctors found 32 studies, but concluded that further research was needed to clarify whether breaks are beneficial [12]. This review also highlighted a lack of studies measuring burnout or patient safety perceptions as outcome measures, and identified a need for more studies in primary care physicians [12]. Our findings complement previous qualitative work which has identified breaks as a realistic and beneficial intervention in primary care settings [11]. They also complement previous cross-sectional survey studies in hospital doctors, which indicate that taking breaks is associated with lower work stress and faster reaction times [12]. More broadly, these results are consistent with a large literature which has linked higher fatigue with higher burnout and poorer patient safety [30, 31]. The present study investigated breaks, a potential antidote to fatigue [32], and highlights breaks as a potential intervention. It also extends this literature by identifying the opportunity for positive interactions as an important feature of effective breaks.

### Implications for research and practice

Having a break during the day where GPs can interact with their colleagues may help improve wellbeing, alleviate burnout and improve patient safety. While this solution may not be the ultimate answer to current staffing shortages, it is practical, feasible, and could be more assertively and routinely implemented with relatively little difficulty compared to alternative solutions. These findings have relevance for healthcare managers; the finding that the opportunity for positive social interactions was important suggests that breaks should be organised at the practice or team level, so staff can take them together.

### Conclusion

Identifying effective and actionable interventions which can help reduce staff burnout is a current priority for healthcare services. Organising practice or team breaks during the working day may help to both reduce GP burnout and enhance patient safety perceptions in primary care.

## Supporting information

S1 Data (DOCX)

S1 Methods Daily measurement of positive and negative affect. (DOCX)

S2 Methods Further information regarding data preparation and analysis. (DOCX)

S1 Table Descriptives for daily Level 1 data, at work only. (DOCX)

S2 Table Total number of breaks and patient safety incidents from Level 1 data, same day analyses. (DOCX)

S1 File (DOCX)
