# Supplementary material for: Could breaks reduce general practitioner burnout and improve safety? A daily diary study
Source: PLoS One. 2024 Aug 27;19(8):e0307513. doi: 10.1371/journal.pone.0307513 (PMC11349094; doi:10.1371/journal.pone.0307513)
Supplement: S1 Data — (DOCX) [file pone.0307513.s001.docx]

**Supplement**

**SMethods 1**

**Daily measurement of positive and negative affect**

Participants rated the extent to which they felt each mood that day, from 0 (‘not at all’) to 10 (‘very much so’). The positive affect score was the cumulative of six items (‘‘happy’, ‘successful’, ‘satisfied’, ‘excited’, ‘capable’, and ‘calm’), (Cronbach’s α = .909) and the negative affect score comprised eight items (‘sad’, ‘anxious’, ‘defeated’, ‘lonely’, ‘guilty’, ‘hopeless’, ‘irritable’, and ‘stressed’), (Cronbach’s α= .922) showing very good internal consistency for the dataset where n = 241. These adjectives were developed for this study with adjectives taken from 1) the Positive and Negative Affect Schedule^1^, 2) Diener and Emmon’s affect scale^2^, and 3) generated due to their specific relevance to this study (successful, satisfied, capable, calm, defeated, lonely, hopeless). This approach is consistent with other similar studies^3^. Factor analysis was conducted within our sample and confirmed the presence of two different factors (one for negative affect, one for positive affect).

**SMethods 2**

**Further information regarding data preparation and analysis**

Age and years in practice were grand centred, gender was uncentred because it was dichotomous.

For the main effect of e.g. breaks on burnout (controlling for age, gender, and number of years in practice), the following equation was used:

Outcome variable (e.g. burnout) = β_00_ + β_01_ (age) + β_02_ (gender) + β_03_ (years in practice) + β_10_ (predictor variable e.g. breaks) + ε

Whereby:

β_00_ = Mean level of outcome variable (e.g. breaks)

β_01 - 03_ = Indicates the extent to which this average is influence by age/gender/years in practice

β_10_ = Indicated the extent to which this average is influence by level of the predictor variable (e.g. breaks).

ε = Error term

For models with significant predictors, additional terms were added to the model to control for baseline traits related to the outcome measure e.g. baseline burnout, baseline safety perceptions). For analyses involving outcome variables that were dichotomous (PSI, AE, NM), the level 1 models were replaced by Bernoulli models.

The data was screened for outliers using boxplots and missing data analyses were run. For level 2 (baseline measures) data, Little’s MCAR test showed that the data were missing completely at random (X^2^ = 146.575, df = 136, *p* = .253). Missing data in this level for each variable ranged from 0 % missing (majority of variables) to 1.7% (OLBI:E3, OLBI:E7, OLBI:D8, PSS2). Missing data in level 2 were replaced with the column mean. For level 1 data, Little’s MCAR test showed that the data were not missing completely at random (X^2^ = 347.105, df = 271, *p* = .001). Missing data in this level for each variable ranged from 4% missing (OLBI:D3, OLBI:E2, OLBI:E3, Safe Practitioner, Happy, Satisfied, Excited, Capable, Lonely, Guilty, Stressed, Adverse Events, Near Misses) to 5.6% (Morning Stress). Missing data in level 1 were replaced with the person mean for that item. Although Little’s test showed the data were not missing at random, because there was no obvious theoretical basis for why there would be a pattern to the missing data, coupled with the small percentage of missing data overall, it was decided that dealing with the missing data by column and person means was acceptable and pragmatic, to maintain enough power to detect effects.

Sensitivity analyses were run on the same day analyses using the original data files with missing data. In HLM, level-1 missing data was deleted when running each analyses, resulting in a maximum of 222 days, a minimum of 216, with 54 participants (as there were 54 complete level 2 cases). The majority of main effects were unchanged by the use of the un-imputed dataset, however it was found that having a break (of any sort) in the day was associated with lower levels of overall burnout. There were no other significant differences.

For all analyses with continuous outcome variables, the estimation of fixed effects with robust standard errors are reported. For analyses with dichotomous outcomes variables and therefore where Bernoulli distribution was used, the estimation of fixed effects for the unit-specific models with robust standard errors are reported.

For continuous models: pseudo R^2^ = (σ^2^F – τ^2^0) / σ^2^F

For Bernoulli models: pseudo R^2^dicho = σ^2^F / (σ^2^F + τ^2^0 + σ^2^R)

Whereby σ^2^F = variance explained by the unconditional model

τ^2^0 = variance explained by the conditional model

σ^2^R = fixed to 3.29 for logistic models

**STable 1: Descriptives for daily Level 1 data, at work only**

241 cases/days (58 participants), average of 4 working days each. Prior to missing data imputation

|  | **Same Day Analyses** *(n=234 to 241)* | | **Lagged Analyses** *(n=128)* | |
| --- | --- | --- | --- | --- |
| **Variable** | **Mean (s.d.)** | **Range** | **Mean (s.d.)** | **Range** |
| Safe Practice^a^ | 2.59 (0.98) | 1 – 05 | 2.57 (1.30) | 1 - 06 |
| Exhaustion ^b^ | 7.67 (2.09) | 3 – 12 | 7.38 (2.07) | 3 - 12 |
| Disengagement ^c^ | 6.97 (1.77) | 3 – 12 | 6.66 (1.79) | 3 - 10 |
| Burnout Overall ^d^ | 14.60 (3.40) | 6 – 24 | 14.04 (3.44) | 6 - 22 |
| Positive Mood ^c^ | 30.97 (10.98) | 5 – 59 | 33.23 (11.68) | 5 - 29 |
| Negative Mood ^d^ | 22.15 (17.23) | 0 - 75 | 20.52 (16.93) | 0 – 75 |
| Happy^a^ | 5.80 (2.06) | 0 – 10 |  |  |
| Successful^f^ | 5.46 (2.13) | 0 – 10 |  |  |
| Satisfied^a^ | 5.26 (2.21) | 0 – 10 |  |  |
| Excited^a^ | 3.19 (2.49) | 0 – 10 |  |  |
| Capable^a^ | 6.05 (1.97) | 0 – 10 |  |  |
| Calm ^b^ | 5.23 (2.31) | 0 – 10 |  |  |
| Sad ^b^ | 2.53 (2.59) | 0 – 10 |  |  |
| Anxious^f^ | 3.53 (2.79) | 0 – 10 |  |  |
| Defeated ^b^ | 2.48 (2.70) | 0 – 10 |  |  |
| Lonely^a^ | 2.05 (2.52) | 0 – 10 |  |  |
| Guilty^a^ | 1.85 (2.34) | 0 – 10 |  |  |
| Hopeless^f^ | 1.84 (2.44) | 0 – 10 |  |  |
| Irritable^f^ | 3.45 (2.98) | 0 - 10 |  |  |
| Stressed^a^ | 4.49 (2.90) | 0 - 10 |  |  |

^a^*n* = 241, ^b^*n* = 240, ^c^*n* = 238, ^d^*n* = 237, ^e^*n* = 234, ^f^*n* = 239

**STable 2: Total number of breaks and patient safety incidents from Level 1 data, Same day analyses**

| **Variable** | **Number (% of cases)** | **Number (% of cases)** |
| --- | --- | --- |
| Breaks | None = 76 (31.5%) | One or more = 165 (68.5%) |
| Positive Interaction | None = 106 (44.0%) | One or more = 135 (56.0%) |
| PSI | None = 201 (83.4%) | One or more = 40 (16.6%) |

241 cases/days (58 participants), average of 4 working days each. Prior to missing data imputation. PSI = Patient Safety Incident (Adverse Event and/or Near Miss)

**SReferences**

1. Watson D, Clark LA, Tellegen A. Development and validation of brief measures of positive and negative affect: The PANAS scales. *Journal of Personality and Social Psychology* 1988;54(6):1063-70.

2. Diener E, Emmons RA. The independence of positive and negative affect. *Journal of personality and social psychology* 1984;47(5):1105.

3. Myin-Germeys I, Delespaul PA, DeVries MW. Schizophrenia patients are more emotionally active than is assumed based on their behavior. *Schizophrenia Bulletin* 2000;26(4):847-54.
