# Supplementary material for: Could breaks reduce general practitioner burnout and improve safety? A daily diary study
Source: PLoS One. 2024 Aug 27;19(8):e0307513. doi: 10.1371/journal.pone.0307513 (PMC11349094; doi:10.1371/journal.pone.0307513)
Supplement: S1 Methods — (DOCX) [file pone.0307513.s002.docx]

**SMethods 1**

**Daily measurement of positive and negative affect**

Participants rated the extent to which they felt each mood that day, from 0 (‘not at all’) to 10 (‘very much so’). The positive affect score was the cumulative of six items (‘‘happy’, ‘successful’, ‘satisfied’, ‘excited’, ‘capable’, and ‘calm’), (Cronbach’s α = .909) and the negative affect score comprised eight items (‘sad’, ‘anxious’, ‘defeated’, ‘lonely’, ‘guilty’, ‘hopeless’, ‘irritable’, and ‘stressed’), (Cronbach’s α= .922) showing very good internal consistency for the dataset where n = 241. These adjectives were developed for this study with adjectives taken from 1) the Positive and Negative Affect Schedule^1^, 2) Diener and Emmon’s affect scale^2^, and 3) generated due to their specific relevance to this study (successful, satisfied, capable, calm, defeated, lonely, hopeless). This approach is consistent with other similar studies^3^. Factor analysis was conducted within our sample and confirmed the presence of two different factors (one for negative affect, one for positive affect).
