# Supplementary material for: Could breaks reduce general practitioner burnout and improve safety? A daily diary study
Source: PLoS One. 2024 Aug 27;19(8):e0307513. doi: 10.1371/journal.pone.0307513 (PMC11349094; doi:10.1371/journal.pone.0307513)
Supplement: S1 Table — (DOCX) [file pone.0307513.s004.docx]

**STable 1: Descriptives for daily Level 1 data, at work only**

241 cases/days (58 participants), average of 4 working days each. Prior to missing data imputation

|  | **Same Day Analyses** *(n=234 to 241)* | | **Lagged Analyses** *(n=128)* | |
| --- | --- | --- | --- | --- |
| **Variable** | **Mean (s.d.)** | **Range** | **Mean (s.d.)** | **Range** |
| Safe Practice^a^ | 2.59 (0.98) | 1 – 05 | 2.57 (1.30) | 1 - 06 |
| Exhaustion ^b^ | 7.67 (2.09) | 3 – 12 | 7.38 (2.07) | 3 - 12 |
| Disengagement ^c^ | 6.97 (1.77) | 3 – 12 | 6.66 (1.79) | 3 - 10 |
| Burnout Overall ^d^ | 14.60 (3.40) | 6 – 24 | 14.04 (3.44) | 6 - 22 |
| Positive Mood ^c^ | 30.97 (10.98) | 5 – 59 | 33.23 (11.68) | 5 - 29 |
| Negative Mood ^d^ | 22.15 (17.23) | 0 - 75 | 20.52 (16.93) | 0 – 75 |
| Happy^a^ | 5.80 (2.06) | 0 – 10 |  |  |
| Successful^f^ | 5.46 (2.13) | 0 – 10 |  |  |
| Satisfied^a^ | 5.26 (2.21) | 0 – 10 |  |  |
| Excited^a^ | 3.19 (2.49) | 0 – 10 |  |  |
| Capable^a^ | 6.05 (1.97) | 0 – 10 |  |  |
| Calm ^b^ | 5.23 (2.31) | 0 – 10 |  |  |
| Sad ^b^ | 2.53 (2.59) | 0 – 10 |  |  |
| Anxious^f^ | 3.53 (2.79) | 0 – 10 |  |  |
| Defeated ^b^ | 2.48 (2.70) | 0 – 10 |  |  |
| Lonely^a^ | 2.05 (2.52) | 0 – 10 |  |  |
| Guilty^a^ | 1.85 (2.34) | 0 – 10 |  |  |
| Hopeless^f^ | 1.84 (2.44) | 0 – 10 |  |  |
| Irritable^f^ | 3.45 (2.98) | 0 - 10 |  |  |
| Stressed^a^ | 4.49 (2.90) | 0 - 10 |  |  |

^a^*n* = 241, ^b^*n* = 240, ^c^*n* = 238, ^d^*n* = 237, ^e^*n* = 234, ^f^*n* = 239
