# Supplementary material for: Could breaks reduce general practitioner burnout and improve safety? A daily diary study
Source: PLoS One. 2024 Aug 27;19(8):e0307513. doi: 10.1371/journal.pone.0307513 (PMC11349094; doi:10.1371/journal.pone.0307513)
Supplement: S2 Table — (DOCX) [file pone.0307513.s005.docx]

**STable 2: Total number of breaks and patient safety incidents from Level 1 data, Same day analyses**

| **Variable** | **Number (% of cases)** | **Number (% of cases)** |
| --- | --- | --- |
| Breaks | None = 76 (31.5%) | One or more = 165 (68.5%) |
| Positive Interaction | None = 106 (44.0%) | One or more = 135 (56.0%) |
| PSI | None = 201 (83.4%) | One or more = 40 (16.6%) |

241 cases/days (58 participants), average of 4 working days each. Prior to missing data imputation. PSI = Patient Safety Incident (Adverse Event and/or Near Miss)
