# Supplementary material for: Could breaks reduce general practitioner burnout and improve safety? A daily diary study
Source: PLoS One. 2024 Aug 27;19(8):e0307513. doi: 10.1371/journal.pone.0307513 (PMC11349094; doi:10.1371/journal.pone.0307513)
Supplement: S1 File — (DOCX) [file pone.0307513.s006.docx]

**SReferences**

1. Watson D, Clark LA, Tellegen A. Development and validation of brief measures of positive and negative affect: The PANAS scales. *Journal of Personality and Social Psychology* 1988;54(6):1063-70.

2. Diener E, Emmons RA. The independence of positive and negative affect. *Journal of personality and social psychology* 1984;47(5):1105.

3. Myin-Germeys I, Delespaul PA, DeVries MW. Schizophrenia patients are more emotionally active than is assumed based on their behavior. *Schizophrenia Bulletin* 2000;26(4):847-54.
